# Maternal cell-free DNA in early pregnancy for preeclampsia screening: a systematic review

**DOI:** 10.1007/s00404-024-07905-4

**Published:** 2025-01-27

**Authors:** Svitlana Arbuzova, Howard Cuckle

**Affiliations:** 1 Eastern-Ukrainian Center for Medical Genetics and Prenatal Diagnosis, Mariupol, Kiev Ukraine; 2 Institute of Health Research, University of Exeter, Exeter, UK; 3 Faculty of Medicine and Health Sciences, Tel Aviv University, Tel Aviv, Israel

**Keywords:** Preeclampsia, Cell-free DNA, Maternal blood, Screening, Markers

## Abstract

**Purpose:**

To quantify the separation between maternal blood cell-free (cf)DNA markers in preeclampsia and unaffected pregnancies and compare with existing markers. This approach has not been used in previous studies.

**Methods:**

Comprehensive systematic literature search of PubMed to identify studies measuring total cfDNA, fetal cf(f)DNA or the fetal fraction (FF) in pregnant women. Included—studies of asymptomatic pregnancies with subsequent preeclampsia (cases) and unaffected pregnancies (controls) tested in the first or second trimester and before the clinical onset of preeclampsia. Excluded—studies not reporting the median or mean, standard deviation, inter-quartile range or range in cases and controls. Information from 26 eligible studies was entered into a meta-analysis to estimate, for each marker, the Mahalanobis distance, a measure of separation between the overlapping distributions in preeclampsia and unaffected pregnancies. This was compared with estimates for mean arterial pressure (MAP), uterine artery Doppler pulsatility index (UtA-PI), pregnancy associated plasma protein (PAPP)-A and placental growth factor (PlGF).

**Results:**

The mean Mahalanobis distance for total cfDNA was 0.44 (95% CI 0.12–0.76), which fell between UtA-PI (0.53) and the absolute value of PAPP-A (– 0.36). For cffDNA the distance was 1.03 (0.37–1.69), which is superior to MAP (0.74), UtA-PI, PlGF (– 0.57) and PAPP-A. The distance for FF was – 0.34 (– 0.56 to – 0.12), similar to PAPP-A.

**Conclusion:**

All three markers have a potential preeclampsia screening role, particularly cffDNA. However, to estimate the screening performance in combination with existing markers further large studies are needed. The current analysis will help in the power calculation for such studies.

## Introduction

Preeclampsia (PE) prevalence can be substantially reduced through first trimester multi-marker screening and aspirin prophylaxis in screen positives. Currently, potential screening markers include mean arterial pressure (MAP), uterine artery Doppler pulsatility index (UtA-PI), maternal serum pregnancy associated plasma protein (PAPP)-A and placental growth factor (PlGF). A protocol including prior risk factors and all four markers has model predicted detection rates of 80% for preterm and 43% term PE, for a false-positive rate of 10% [1]. And the efficacy of aspirin treatment in screen positives has been confirmed by the ASPRE double blind placebo controlled randomized clinical trial [2].

Five literature reviews have considered the possibility of using different cell-free (cf)DNA species as preeclampsia screening markers [3–7]. The species were total cfDNA, fetal cf(f)DNA and the fetal fraction (FF, cffDNA divided by total cfDNA). However, only one review carried out a formal meta-analysis, and this was limited to cffDNA [5]. A simulation model was fitted to estimate the detection rate for a false-positive rate of 10%. When tests were carried out at 15–28 weeks there was a statistically significant estimated detection rate of 37%.

In the current report a further comprehensive systematic review is carried out and meta-analysis is used to estimate the Mahalanobis distance, a measure of separation between affected and unaffected pregnancies, for each cfDNA species. This approach was not used in the previous reviews or the individual studies although it has been employed successfully in the assessment of Down syndrome screening markers [8]. The screening potential of a putative marker is dependent on the separation between the overlapping distributions. A greater separation, and hence lesser overlap, indicates a higher potential. The Mahalanobis distance quantifies the separation as the average distance between affected and unaffected pregnancies expressed in terms of standard deviations.

Here, the Mahalanobis distance is compared with the corresponding estimate for MAP, UtA-PI, PAPP-A and PlGF based on published results. If one or more species were shown to provide separation comparable to the established markers it could be particularly suitable for centres that screen for Down syndrome by cfDNA alone.

Unlike in early pregnancy, third trimester preeclampsia screening, using PlGF and soluble fms-like tyrosine kinase-1 aims to estimate prognosis among women presenting with symptoms [9]. There may also be a role for cfDNA at that gestation but it is outside the scope of this review.

## Methods

### Literature search

A comprehensive systematic literature search of PubMed was carried out to identify studies that measured serum or plasma total cfDNA, fetal cf(f)DNA or the fetal fraction (FF) in blood samples from pregnant women with or without preeclampsia. The keywords used in the search were a combination of “preeclampsia” and one of the following: “cell-free DNA”, “cf-DNA”, “cffDNA”, “fetal DNA”, “maternal DNA”, “total DNA” and “fetal fraction”. All five previous reviews used PubMed; additionally two used Embase, two Web of Knowledge and one Scopus.

#### Inclusion criteria

- Publication up to October 2024
- Blood sampled in the first or second trimester
- Pregnancies subsequently diagnosed with preeclampsia (cases) and unaffected pregnancies (controls)
- Average values, mean or median, included in the publication as well as standard deviations (SD), inter-quartile range (IQR, 25th–75th percentile) or total range

#### Exclusion criteria

Editorial, Letters to Editor and Conference Abstract.

### Mahalanobis distance

For each study the Mahalanobis distance was calculated as the average in cases minus the average in controls divided by the mean of the SD in cases and controls. For those not reporting SD, this was estimated from the IQR by (75th percentile minus 25th percentile)/1.35 or the whole range by (highest minus lowest value)/8. These estimates assume that cfDNA follows a Gaussian distribution in which case they will approximate to a directly measured SD. Some studies divided the results according to the gestation at preeclampsia onset or gestation when the blood sample was drawn. One study reported results for two different cffDNA methods. Mahalanobis distance was calculated separately for each subgroup.

Overall, and for studies with onset and sampling subgroups, the weighted mean Mahalanobis distance was calculated using the weight 1/(1/*n* + 1/*m*), where *n* is the number of cases and *m* is the number of controls. The 95% confidence interval (CI) on the weighted averages was also calculated from the standard error. Analysis of variance was used to determine differences according to analytical method and the Levene test was used to assess homogeneity of variances. All computations were carried out using SAS software (version 9.4; SAS Institute Inc., Cary, North Carolina).

The comparable results for established preeclampsia markers were derived from two large series reported by the King’s College Hospital group [10, 11]. The mean multiple of the median (MoM) was reported for 752 early, intermediate and late onset preeclampsia cases and 32,850 unaffected pregnancies [10]; the standard deviation of log_10_ MoM was reported in 1426 preeclampsia cases and 57,458 unaffected pregnancies [11]. All four existing markers follow a log Gaussian distribution so the Mahalanobis distance was estimated from the log_10_ MoM means, and for preeclampsia the log_10_ value in each onset group was averaged after weighting for the number of cases.

### Adjustment for covariables

In studies that reported results with and without adjustment for potentially confounding covariables, only unadjusted results were included in the meta-analysis.

## Results

The search identified 26 eligible studies [12–37], all but 6 of which were included in the five previous systematic reviews [3–7] and the additional studies were published after 2019, the most recent previous search period [7]. For each study and subgroup, the average total cfDNA, cffDNA and FF in cases and controls, together with the numbers and SD, IQR or range, are shown in Appendix Tables A1–A3. The tables also show the gestational age of onset and blood sampling, and the analytical method.

Eight studies included pregnancies with known male fetuses, determining cffDNA by measuring Y-specific gene fragments such as SRY [12, 13, 16, 19, 24] and DSY14 [14, 20, 27]. Others studied all fetuses, determining separately total cfDNA and cffDNA by measuring genes that are methylated in the fetus and placenta but unmethylated in the mother, such as DSCR3 [28], RASSF1A [15, 21, 22, 28, 34] and HYP2 [28, 32], and RhD [18]. Total cfDNA was also determined by measuring the ubiquitous genes β-globin [19] and β-actin [19, 26], and cffDNA by measuring the placental gene DYS1 [17]. Eight studies used proprietary methods for cfDNA, cffDNA and FF, based on digital analysis of selected regions (DANSR) [23, 35] or shotgun massively parallel sequencing (s-MPS) [26, 30, 31, 33, 36, 37].

In 12 out of 13 studies reporting total cfDNA, the average concentration was higher in the cases compared with their controls and in 21 out of 23 including subgroups. Among 16 studies of cffDNA the average concentration was higher in cases for 14; and 20 out of 24 including subgroups. For all six studies reporting FF the average was lower among cases; and in 13 out 15 including subgroups.

**Fig. 1:**
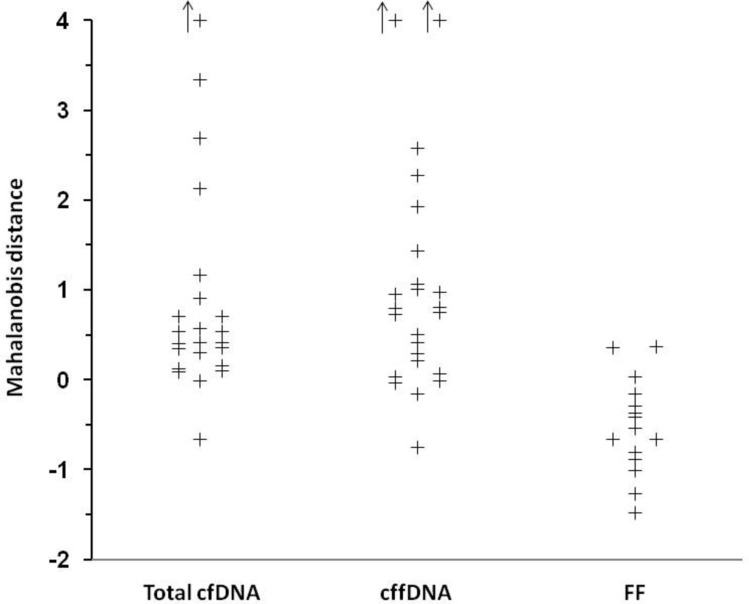
Mahalanobis distance for each individual study and subgroup (upward arrow above 4)

Figure 1 shows the Mahalanobis distance for each individual study and subgroup reporting total cfDNA, cffDNA and FF. Table 1 shows the estimated average distance, overall and for onset or sampling subgroups. The comparable distances for MAP, UtA-PI, maternal serum PlGF and PAPP-A were respectively 0.74, 0.53, – 0.57 and – 0.36.

**Table 1:** Mahalanobis distance: all studies and subgroups according to onset of preeclampsia and sample

Studies & subgroups	Number	Samples		Mahalanobis distance		
		Cases	Controls	Average	Lower 95% CI	Upper 95% CI
Total cfDNA						
All	23	526	1882	0.44	0.12	0.76
Early onset subgroups	4	34	420	0.60	0.29	0.92
Late onset subgroups	5	79	520	0.23	– 0.11	0.57
Earliest sample subgroups	7	73	194	0.64	– 0.28	1.56
Latest sample subgroups	8	103	516	0.77	0.15	1.38
cffDNA						
All	24	596	2289	1.03	0.37	1.69
Early onset subgroups	2	47	313	4.31	0.37	8.25
Late onset subgroups	2	135	313	0.54	– 0.21	1.28
Earliest sample subgroups	5	61	334	0.59	0.04	1.14
Latest sample subgroups	5	65	198	1.44	– 1.09	3.97
FF						
All	15	688	13,871	– 0.34	– 0.56	– 0.12
Early onset subgroups	7	260	12,250	– 0.41	– 0.89	0.07
Late onset subgroups	7	464	12,214	– 0.23	– 0.41	– 0.05
Earliest sample subgroups	6	174	2104	– 0.19	– 0.62	0.25
Latest sample subgroups	5	76	652	– 0.13	– 0.55	0.29

The overall estimate for total cfDNA was 0.44 (95% CI 0.12–0.76), which in absolute terms falls between UtA-PI and maternal serum PAPP-A. For cffDNA the estimate was 1.03 (95% CI 0.37–1.69), which is superior to all the existing markers. The estimate for FF, – 0.34 (95% CI – 0.56 to – 0.12), is similar to maternal serum PAPP-A. There were wide confidence intervals around all three estimates, although the lower limits for total cfDNA and cffDNA, and the upper limit for FF excluded zero (ie no separation between the distributions). For cffDNA, even the lower limit is similar in absolute terms to maternal serum PAPP-A.

The gestation of onset subgroup estimates indicate greater separation between the distributions for early compared to late onset. For total cfDNA 0.60 versus 0.23, for cffDNA 4.31 versus 0.54, and for FF – 0.43 versus – 0.23, respectively. The corresponding sampling subgroup estimates for total cfDNA and cffDNA show less separation in samples drawn earliest: 0.64 versus 0.77 and 0.59 versus 1.44, respectively. In contrast, with FF there were no material differences in Mahalanobis distance between the subgroups.

There were statistically significant differences among the Mahalanobis distances for total cfDNA according to analytical method (*P* < 0.02) and significant heterogeneity in the variances (*P* < 0.05). This was due to the subgroup using RASSF1A which had an average of 1.78 (95% CI 0.77–2.78), while the other methods were not statistically different (*P* = 0.24) and together had an average of 0.22 (95% CI 0.08–0.36). There were no statistically significant method differences for cffDNA (*P* = 0.47) or heterogeneity of variance (*P* = 0.52); for FF there were no statistically significant differences (*P* = 0.59) and insufficient studies to assess heterogeneity of variance.

## Discussion

This is the first report to compare the separation in cfDNA marker distributions between preeclampsia and unaffected pregnancies. There was a statistically significant increase in separation for total cfDNA and cffDNA, and decrease in FF.

The separation was found to be similar, or superior, to existing preeclampsia screening markers. For total cfDNA, the separation fell between that of UtA-PI and maternal serum PAPP-A; for FF it was similar to maternal serum PAPP-A, the weakest existing marker; and for cffDNA it was superior to all existing markers. Nonetheless, despite the relatively large number of results included in the meta-analysis, the Mahalanobis distance confidence intervals were wide, indicating some uncertainly. The confidence limits for all three cfDNA markers exclude the possibility of no screening potential, and the lowest potential for cffDNA is comparable with that of PAPP-A. At the other extreme, the results for cffDNA are consistent with a potential superior to that of all conventional markers.

The literature search only used PubMed, while three of the five previous reviews additionally used Embase [3, 6], Web of Knowledge [5, 6] and Scopus [5]. However, this single source is likely to be complete since it identified all relevant publications found by the three previous multi-source reviews. Moreover, additionally searching Embase after the period of the five reviews did not yield further relevant publications.

Although only a few studies contributed to subgroup analysis the findings indicate greater separation between the distributions for early compared to late onset. This is consistent with the findings for the existing preeclampsia markers. For cfDNA and cffDNA the findings support less separation in samples drawn earliest in pregnancy than later. This is consistent with existing preeclampsia markers where the detection rate for a given false-positive rate steadily increases between the first, second and third trimesters [38].

Most of the 26 studies included in the analysis are under-powered, hence meta-analysis provides the most reliable estimation of Mahalanobis distance. Moreover, particularly for total cfDNA and cffDNA, the individual studies have considerable differences in units of measurement and analytical methods. The use of Mahalabinobis distance standardizes for the units since averages are divided by standard deviations. For total cfDNA there were statistically significant differences according to method attributable solely to those using RASSF1A which yielded a considerably higher Mahalanobis distance than the other methods. The studies also differed in size but this was accounted for in the computation of mean Mahalanobis distance by the use of weighting according to the numbers of cases and controls.

Most of the studies did not take account of covariables which are risk factor for PE [39]. An important potential confounder is increased maternal weight or body mass index (BMI). It is speculated that *maternal* cfDNA is of hematopoietic [40] or adipose tissue [41, 42] origin while cffDNA is of placental origin. If so, confounding might be expected to increase total cfDNA in preeclampsia and reduce the FF; indeed in cfDNA Down syndrome screening programs BMI is an established cause of very low FF [43]. Nonetheless, the relationship between maternal weight may be more complex since a study of mice found that maternal obesity reduces cffDNA release [44].

Only one study stated that they had adjusted results (FF) for maternal factors, and CRL. Four studies reported results with and without adjustment for covariables, although the analysis only includes the unadjusted results. Adjustment was for: CRL, maternal weight, height, ethnicity and in vitro fertilization [27]; maternal age and BMI [28]; maternal age, gestation and BMI [32]; gestation, BMI and smoking [27]. However, adjustment did not completely account for the findings. Mahalanobis distances with and without adjustment were calculated for three of them: total cfDNA 0.44 and 0.29 [26], 0.49 and 0.49 [28], 0.35 and 0.51 [32]; cffDNA 0.48 and 0.48 [28]; FF – 0.56 and – 0.31 [26]. Two other studies did not report actual adjusted results but noted changes in statistical significance after adjustment: maternal age, parity and smoking [19]; and maternal age, ethnicity, BMI, history of chronic hypertension and test method [30]. In one, the non-significant increases in average total cfDNA and cffDNA remained non-significant after adjustment [19] and in the other, the statistically significant reductions in FF at 10–14 weeks in PE with onset ≤ 34 and > 34 weeks were not significant after adjustment [30].

The Malanobis distance meta-analysis shows that cfDNA markers of preeclampsia are comparable to existing markers. This reinforces and expands on other reported findings.

In addition to case-controls studies, there are prospective studies indicating the utility of FF. TRIDENT-2, a prospective study of cfDNA screening for aneuploidy in the Netherlands [45], found that 268 women with FF < 4% had PE prevalence of 4.1% compared with 2.3% in the general Dutch population. This increase was not statistically significant overall but only in term PE. A recent update found that the incidence of hypertensive disorders of pregnancy in those with FF < 2.5% was 9.9% and for FF ≥ 2.5% it was 5.6% [46].

The previous meta-analysis of cffDNA studies estimated two indicators of preeclampsia screening performance: detection rate for a 10% false-positive rate; and the area under the receiver-operator characteristic curve (AUC) [5]. Estimation was from a simulation model based on averages and standard deviations and taking account of BMI differences between cases and controls. In eight studies sampling at 15–28 weeks the detection rate was 37% (95% CI 32–42%) and AUC 0.73 (0.70–0.76); there were only two available studies with earlier testing and the results were not statistically significant.

The authors did not compare the results with established preeclampsia markers but there are published estimates which suggest that cffDNA performance might be comparable. For example, in one report the detection rates for a 10% false-positive rate when testing at 11–13 weeks were, for all cases and those presenting < 34 weeks: UtA-PI 42% (40–45%) and 75% (69–81%); MAP 54% (51–56%) and 73% (67–78%); PlGF 40% (38–43%) and 72% (66–78%); PAPP-A 42% (40–45%) and 55% (48–61%) [47]. AUC values in two reports were: MAP 0.77 (0.77–0.78) [48]; UtA-PI, < 34 and ≥ 34 weeks, 0.83 (0.74–0.91) and 0.63 (0.56–0.69); PlGF 0.80 (0.70–0.89) and 0.65 (0.59–0.71); and, PAPP-A 0.74 (0.64–0.85) and 0.58 (0.51–0.64) [49].

The detection rate for a 10% false-positive rate may appear to be a clinically more direct indicator of potential screening performance than Mahalanobis distance or AUC. However, this is not the case in screening protocols that combine information on risk factors and marker levels. In that context, the potential of a given marker is the *marginal* increase in detection over risk factors alone, rather than absolute detection.

Furthermore, no studies have considered the marginal increase in detection when using cfDNA as an *additional* marker into current preeclampsia screening protocols, though two studies have done this for AUC. None have assessed the changes in detection rate or AUC when *replacing* an existing preeclampsia marker by cfDNA. In one study the AUC for total cfDNA, 0.68, was statistically significant (*P* < 0.05) and maternal serum PAPP-A measured in the same series also had a significant AUC (0.70, *P* < 0.0005) [28]. When both total cfDNA and PAPP-A are considered together the AUC increased to 0.82 (*P* < 0.0001). However, based on the reported confidence intervals, the incremental increase in the AUC for total cfDNA plus PAPP-A compared with PAPP-A alone did not reach statistical significance (*P* = 0.06). In the other study the AUC for maternal factors, UtA-PI, MAP and PAPP-A was 0.91 and did not materially increase when FF was added [35].

Also, when assessing the efficacy of adding or replacing a marker, it is necessary to take into account the correlation between different markers. FF is negatively correlated was MAP and UtA-PI [50], and positively correlated with maternal serum PAPP-A and PlGF [43, 50]. This might be explained to some extent by the presence of PE cases in the cohort but the numbers of such cases is likely to have been relatively small.

Cell-free nucleic acids other than total cfDNA, cffDNA and FF have been reported to be promising markers of preeclampsia. One study demonstrated differences in the cfDNA methylation pattern between cases and controls [36]. The methylome was examined across 200 regions and combined into a score using a regression formula. Among 61 cases presenting < 34 weeks and 136 controls the detection rate for a 10% false-positive rate was 38% and the AUC was 75%. Several studies have reported the potential of cfRNA markers in preeclampsia screening, including messenger RNA, micro RNA and non-coding species (see review [51]).

Future studies must have sufficient power, ideally for both early and late onset PE, and the Mahalanobis distance estimates in this analysis are a guide in calculating power. Centres currently carrying out PE screening might be excluded since intervention will likely bias the outcome. A practical and narrow gestational range should be chosen. Studies should be more statistically advanced and robust than most in this review. Researchers should determine the shape of the marker frequency distribution in affected and unaffected pregnancies; covariables like maternal weight or BMI should be allowed for; and correlation between the cfDNA marker and existing markers will need to be assessed.

In conclusion, Mahalanobis distance analysis provides clarification of cfDNA potential in preeclampsia screening, lending particular support for a role of cffDNA. The analysis overcomes the low statistical power of most individual studies and standardizes for some of the differences in study design. Considerable more focused research is needed before firm conclusions can be drawn on how to combine cfDNA and existing markers.

## Data Availability

No datasets were generated or analyzed during the current study.

## Appendix

See Tables 2, 3, 4.

**Table 2:** Total cfDNA concentration in 13 studies

Study (first author, year)	PE onset (wks)	Blood sample (wks)	Method	Units	Average total cfDNA (*n*) [± SD], [IQR] or [[range]]	
					PE	OK
Farina, 2004 [15]	All	2T	β-globin	GE/mL	439 (8) [± 299]	284 (40) [± 138]
Crowley, 2007 [19]	All	7–20	β-actin	GE/mL	2025 (16) [[250–9950]]	1835 (72) [[26–19500]]
Papantonio, 2013 [21]^a^	All	11–13	RASSF1A	GE/mL	9402 (24) [7281–12432]	2698 (48) [1637–4971]
Kim, 2013 [22]	All	7–14	RASSF1A	C/mL	26,289 (4) [± 8924]	4007 (36) [± 537]
	All	15–28	RASSF1A	C/mL	7962 (5) [± 2530]	3254 (78) [± 288]
Poon, 2013 [23]^b^	All	11–13	DANSR	GE/mL	151 (46) [109–221]	128 (1085) [90–182]
Salvianti, 2015 [25]	All	6–16	RASSF1A	GE/mL	290 (3) [[113–313]]	282 (13) [[15–795]]
	All	17–23	RASSF1A	GE/mL	770 (6) [[102–1033]]	311 (14) [[71–1867]]
Rolnik, 2015 [26]	< 34	11–13	s-MPS	GE/mL	2104 (20) [1454–3547]	1590 (200) [1111–2312]
	≥ 34	11–13	s-MPS	GE/mL	2178 (20) [1123–2847]	
	≥ 34	20–24	s-MPS	GE/mL	2140 (20) [1067–2934]	1746 (100) [1162–2311]
Kim, 2016 [28]	All	6–14	HYP2	C/mL	7170 (14) [4895–12384]	5188 (53) [2043–7682]
	All	15–23	HYP2	C/mL	11,262 (20) [9416–16781]	8505 (31) [5795–11489]
Silver, 2017 [29]	All	9–12	β-actin	NR	3.52 (175) [0.11–25.3]	3.74 (175) [0.12–21.14]
Yuan, 2019 [31]	All	12–22	s-MPS	ng/mL	6.42 (52) [4.94–9.35]	5.99 (630) [4.58–7.49]
Kwak, 2020 [32]	Early	2T	HYP2	C/mL	22,394 (6) [14992–38659]	9441 (78) [5878–12251]
	Late	2T	HYP2	C/mL	10,023 (23) [6356–16374]	
Karapetian, 2021 [34]	All	11–14	RASSF1A	GE/mL	732 (20) [442–1700]	208 (22) [157–461]
	All	24–26	RASSF1A	GE/mL	753 (20) [556–3186]	299 (22) [127–459]
Gekas, 2023 [37]^c^	< 34	11–14	s-MPS	ng/mL	58 (4) [34–121]	37 (71) [31–50]
	≥ 34	11–14	s-MPS	ng/mL	26 (8) [22–48]	
	< 34	17–25	s-MPS	ng/mL	40 (4) [30–60]	29 (71) [23–35]
	≥ 34	17–25	s-MPS	ng/mL	34 (8) [28–54]	

*2T* second trimester, *DANSR* digital analysis of selected regions, *s-MPS* shotgun massively parallel sequencing, *GE* genome equivalents, *C* copies

^a^IQRs not reported but derived here approximately from a figure

^b^Average maternal and fetal values not reported separately and total cfDNA here was calculated by adding the reported maternal and fetal values

^c^Averages and IQRs not reported but derived here approximately from a figure

**Table 3:** cffDNA concentration in 16 studies

Study (first author, year)	PE onset (wks)	Blood sample (wks)	Method	Units	Average cffDNA (*n*) [± SD], [IQR] or [[range]]	
					PE	OK
Male fetuses						
Leung, 2001 [12]	All	11–22	SRY	GE/mL	41.9 (18) [25.8–62.8]	22.0 (33) [15.3–31.5]
Zhong, 2002 [13]	All	19–25	SRY	C/mL	423 (10) [[97.3–1642]]	128 (40) [[30.6–318]]
Farina, 2004 [14]	All	18–24	DSY14	MoM	2.39 (6) [± 2.84]	1.00 (30) [± 0.86]
Cotter, 2004 [16]^a^	All	mn = 16	SRY	C/mL	25,119 (88) [12589–125892]	5012 (176) [0–15849]
Crowley, 2007 [19]	All	7–20	SRY	GE/mL	30.5 (16) [[0–214]]	27.5 (72) [[0–1280]]
Sifakis, 2009 [20]	< 34	11–13	DSY14	GE/mL	95.5 (11) [72.7–140.9]	51.5 (176) [31.1–84.9]
	≥ 34	11–13	DSY14	GE/mL	50.8 (33) [25.0–103.8]	
Yu, 2013 [24]	< 34	2T	SRY	NR	1202 (20) [912–4786]	61.6 (20) [28.8–339]
Thurik, 2016 [27]	All	8–13	DSY14	GE/mL	54.6 (37) [36.6–66.7]	49.4 (96) [33.1–66.7]
All fetuses						
Levine, 2004 [17]	< 37	2T	DYS1	GE/mL	74 (36) [± 15]	16 (137) [± 6]
	≥ 37	2T	DYS1	GE/mL	22 (102) [± 9]	
Cotter, 2005 [18]^b^	All	9–22	RhD	C/mL	8660 (23) [10000–13182]	3981 (23) [2344–14125]
Papantonio, 2013 [21]^a^	All	11–13	RASSF1A	GE/mL	934 (24) [791–1720]	62 (48) [27–133]
Kim, 2013 [22]	All	7–14	RASSF1A	C/mL	120 (4) [± 52.2]	60.2 (36) [± 9.7]
	All	15–28	RASSF1A	C/mL	189 (5) [± 24.2]	60.1 (78) [± 5.7]
Poon, 2013 [23]	All	11–13	DANSR	GE/mL	12.9 (46) [9.9–20.9]	13.3 (1085) [10.1–17.7]
Salvianti, 2015 [25]	All	6–16	RASSF1A	GE/mL	0.65 (3) [[0–32]]	1.15 (13) [[0–20]]
	All	17–23	RASSF1A	GE/mL	1.95 (6) [[0–32]]	4.43 (14) [[0–21]]
Kim, 2016 [28]	All	6–14	DSCR3	C/mL	939 (14) [686–1718]	891 (53) [539–1530]
	All	6–14	RASSF1A	C/mL	669 (20) [446–962]	446 (31) [263–936]
	All	15–23	DSCR3	C/mL	2594 (14) [2113–3002]	1780 (53) [1444–2591]
	All	15–23	RASSF1A	C/mL	1653 (20) [1068–2314]	1334 (31) [728–2403]
Karapetian, 2021 [34]	All	11–14	RASSF1A	GE/mL	54.85 (20) [29.10–131.8]	14.15 (22) [6.55–19.40]
	All	24–26	RASSF1A	GE/mL	96.72 (20) [35.11–251.4]	24.87 (22) [15.61–38.80]

*2T* second trimester, *mn* mean, *DANSR* digital analysis of selected regions, *GE* genome equivalents, *C* copies, *NR* not reported, *MoM* case & five matched controls divided by median control

^a^IQRs not reported but derived here approximately from a figure

^b^Averages and IQRs not reported but derived here from a figure

**Table 4:** Average fetal fraction (%) in six studies, all using the s-MPS method

Study (first author, year)	PE onset (wks)	Blood sample (wks)	Method	Units	Average fetal fraction (*n*) [± SD] or [IQR]	
					PE	OK
Rolnik, 2015 [26]	< 34	11–13	s-MPS	%	6.85 (20) [6.29–7.81]	8.74 (200) [6.73–11.0]
	≥ 34	11–13	s-MPS	%	7.69 (20) [6.49–9.83]	
	≥ 34	20–24	s-MPS	%	8.20 (20) [5.70–10.7]	9.65 (100) [7.60–12.0]
Bender, 2019 [30]	≤ 34^b^	10–14	s-MPS	%	10.4 (61) [± 4.94]	12.4 (1833) [± 5.88]
	> 34^b^	10–14	s-MPS	%	10.8 (61) [± 5.20]	
	≤ 34^b^	14–20	s-MPS	%	14.6 (19) [± 7.78]	12.1 (481) [± 5.92]
	> 34^b^	14–20	s-MPS	%	12.3 (25) [± 5.15]	
Yuan, 2020 [31]	All	13–26	s-MPS	%	8.49 (64) [6.49–11.74]	11.08 (1521) [8.40–13.71]
Sapantzoglou, 2022 [35]	< 37	11–13	DANSR	MoM	0.825 (91) [0.689–1.115]	1.002 (9458) [0.785–1.251]
	≥ 37	11–13	DANSR	MoM	0.946 (222) [0.728–1.211]	
De Borre, 2023 [36]	< 34	10–15	s-MPS	%	6.20 (61) [5.20–8.00]	9.60 (136) [7.57–12.0]
Gekas, 2023 [37]^a^	< 34	11–14	s-MPS	%	7.6 (4) [5.3–9.3]	10.0 (71) [6.8–12.6]
	≥ 34	11–14	s-MPS	%	5.3 (8) [4.6–7.4]	
	< 34	17–25	s-MPS	%	7.9 (4) [6.1–9.4]	11.0 (71) [8.5–13.5]
	≥ 34	17–25	s-MPS	%	8.3 (8) [5.3–9.3]	

*s-MPS* shotgun massively parallel sequencing, *DANSR* digital analysis of selected regions, *MoM* multiple of the expected median based on maternal factors and CRL

^a^Averages and IQRs not reported but derived from a figure

^b^Severe PE
